# The rise of *‘*smart’ solutions in Africa: a review of the socio-environmental cost of the transportation and employment benefits of ride-hailing technology in Ghana

**DOI:** 10.1057/s41599-022-01258-6

**Published:** 2022-07-25

**Authors:** Festival Godwin Boateng, Samuelson Appau, Kingsley Tetteh Baako

**Affiliations:** 1 Centre for Sustainable Urban Development, Columbia Climate School/Earth Institute, Columbia University, New York, USA; 2 Melbourne Business School, 200 Leicester Street, Carlton, VIC 3053 Australia; 3 School of Property, Construction and Project Management, RMIT University, Melbourne, Australia

**Keywords:** Science, technology and society, Sociology, Development studies

## Abstract

Governments in Africa are licensing major global ride-hailing firms to launch operations in the continent. This is often presented as a refreshing development for the continent to leverage technology to address its twin problems of inefficient urban transport and rising youth unemployment. Interviews with ride-hailing adopters (drivers, riders, and car owners) and researchers in Ghana suggest, however, that whereas the technology is driving up the standards of road transport experience, the benefits are accessible to a select few (largely, the younger, highly educated and relatively high income-earning class). The lopsided power relations underlying the ride-hailing industry have also meant that the economic opportunities it avails disproportionately benefit a few powerful players (e.g. ride-hailing firms and car owners) while stimulating *‘turf wars’* among online and traditional taxi drivers; deepening existing gender inequalities in access to income-earning opportunities in the commercial passenger transport sector; encouraging unhealthy driving practices, shifts from shared public transport, and inundation of the roads with more private cars. While it will be imprecise to say that the private gains of ride-hailing outstrip the public costs and, therefore, the technology is detrimental to Ghana’s development, the considered evidence raises the need for sustained scrutiny of the hailing of technological interventions as though they are the magic bullets for socio-economic transformation in Africa. Overall, the paper argues that dismantling the power structures underlying Africa’s urban challenges will require more than splashing *‘smart’* apps and other tech wizardries around. Indeed, the lessons from Ghana’s ride-hailing industry suggest that such exclusively technical solutions could easily take root and pattern after existing strictures of unjust power structures in ways that could exacerbate the social and environmental problems they are supposed to address.

## Introduction

Africa is one of the important sites of global urbanization; the continent is projected to accommodate a significant percentage of the over 2.5 billion people set to join the world’s population by the year 2050 (Boateng, 2019; 2021a; United Nations, 2014). It is also one of the concerning sites of global poverty and unemployment (see e.g., Chakravorti and Chaturvedi, 2019). Some 41% of the population lives in poverty, meanwhile, the continent’s fast-growing youthful labor force continues to struggle to enter employment and find a livelihood (Fox and Gandhi, 2021; World Bank, 2020). To worsen matters, the road transport sector, the main means of mobility in the continent, meant to connect people to opportunities too is inefficient and unsafe. For instance, Africa’s rate of road traffic deaths is the worst in the world: 26.6 deaths per 100,000 people (Boateng, 2020; WHO, 2018). The United Nations Environment Program (UNEP) estimates that air pollution, to which vehicular emission contributes significantly, kills 600,000 people every year on the continent (UNEP, 2016; Bonsu et al., 2020).

Research (e.g. Agyemang, 2020; Acheampong, 2021; Delaunay, 2021; Dzisi et al., 2020, 2021; Kaye-Essien, 2020; Kufuor, 2018) and media reports (e.g. Akwagyiram, 2019; Myjoyonline, 2018; Graphic Online, 2017; 2019a; Modern Ghana, 2018) suggest that governments in Africa are granting licenses to major global ride-hailing firms including Uber and Bolt (formerly known as Taxify) to launch operations in the continent. Their operations are even bolstering the emergence of several relatively smaller indigenous ride-hailing firms that also are providing on-demand ride-hailing services in cities on the continent. Considering the frequent admonition that Africa should embrace digital technology to ‘leapfrog’ ahead in its economic development (see e.g., Chakravorti and Chaturvedi, 2019; Echendu and Okafor, 2021), the growing embracement of ride-hailing technology on the continent is a refreshing development.

Ride-hailing—built on the idea of sharing economy—the web-based promotion/coordination of flexible access to—instead of ownership of—underused goods and services—is said to enable both private *vehicle sharing* (one or a group of passengers enjoys and pays for the ride exclusively—e.g. UberX and Lyft), and *passenger ride* or *trip sharing* (unacquainted passengers with similar origins and destinations share a ride and split the fare—e.g. UberPool and Lyft Shared). By facilitating *vehicle* and *passenger-rides* sharing, on-demand ride-hailing is seen as a promising platform for boosting efficient and flexible use of private vehicles by providing *‘a need-basis’* access to—instead of ownership of–private vehicles (Alonso-Moraa et al., 2017; Cohen and Kietzmann, 2014; Fulton et al., 2017; Jin et al., 2018; Santi et al., 2014; Vazifeh et al., 2018). Ultimately, consumer behavior will be altered towards possessing fewer private cars, and overall reduction in the number of cars on roads and lead to increased public health benefits in reduced congestion, energy consumption, greenhouse gas (GHG) emissions, and car crashes.

Additionally, on-demand ride-hailing is considered a source of paid work (Hall and Krueger, 2016; Jin et al., 2018; Camp, 2017; Woodcock and Graham, 2020). For instance, in 2017, Uber co-founder Garrett Camp wrote that the company provides flexible work options to 2 million drivers (see Camp, 2017). Some recent estimates put the number at 3.9 million drivers (see, for instance, Woodcock and Graham, 2020, p. 51).

Viewed this way, ride-hailing firms in Africa can be expected to promote flexible, efficient, and sustainable use of private vehicles while creating paid jobs and other economic opportunities in the cities. Thus, ride-hailing technology could potentially help the continent to address its twin problems of inefficient urban transport and rising youth unemployment, without undermining the environment.

The challenge, however, is that some technologists have begun questioning the adequacy of so-called *‘smart’* apps and other similar digital interventions as tools for resolving complex societal problems (see e.g., Green, 2019; Greenfield, 2013; Zhongming et al., 2020, Chapter 6). They warn that not only do such exclusively technical/technological solutions tend to produce significant unintended adverse consequences; but they also seldom target the power structures that often underlie societal problems. More fundamentally, the benefits of technology transfer are seldom automatic. It is well-documented that when technologies free themselves from their context of discovery and spread to contexts with different socio-cultural and political–economic conditions, they shape and are often, in turn, shaped by the socio-cultural political–economic structures of the recipient societies (Luper et al., 1991; Molnar, 1978; Samli, 1985 in Kebede et al., 2013).

The resulting transformation could either enhance or undermine the viability of the technology. The rapid expansion of ride-hailing technology into Africa, therefore, needs to be carefully studied with the view to learning how they are transforming and are being transformed, in turn, by the local socio-cultural political economy structures and dynamics of the continent’s transport sectors. This will help to properly gauge the potential of the technology to support the generation of decent paid jobs and sustainable use of private cars in the continent. This paper considers the case of Ghana–one of the West African countries experiencing increased presence of ride-hailing firms in its transport sector. In July 2016, the Government of Ghana signed a *‘Statement of Understanding’* (SOU) with Uber to enable the company to launch operations in the country. Today, research (Agyemang, 2020; Acheampong, 2021; Kaye-Essien, 2020; Dzisi et al., 2020, 2021; Kufuor, 2018) and media reports (see e.g., Myjoyonline, 2018; Graphic Online, 2017, 2019a; Modern Ghana, 2018) suggest that other global giants including Bolt and some relatively smaller ride-hailing firms too have begun providing ride-hailing services in the country—particularly in Accra, the capital, and Kumasi, the second biggest city.

As of 2018, Uber alone was said to have about 180,000 active riders in Ghana and some 3000 driver-partners as of 2016 (Dahir, 2017). Ghana’s ride-hailing industry, though rapidly expanding, has remained under-researched. The existing studies have focused on issues such as consumers’ adoption factors and use patterns (e.g., Acheampong et al., 2020; Ofori et al., 2021); the demographic characteristics of people who most patronize the services (Dzisi et al., 2020) and the so-called *‘turf war’* between online and orthodox taxi drivers (e.g., Agyemang, 2020; Kufuor, 2018). Other studies have explored Ghanaians’ perception of the security and safety issues with ride-hailing (Acheampong, 2021); the (in)adequacy of existing transport laws/institutions for regulating the industry (Agyemang, 2020; Kufuor, 2018), and recently, the impact of the Covid-19 pandemic on patronage (Dzisi et al., 2021). This study explores how the operations of ride-hailing firms are transforming and being transformed, in turn, by the local socio-cultural political economy structures of Ghana’s transport sector and what the outcomes mean for ride-hailing potential to support sustainable use of private cars and access to decent paid jobs in the country.

The paper’s main research question thus examines the costs and benefits of how ride-hailing firms have transformed and, in turn, been transformed by the contextual dynamics of Ghana’s transport sector. In addressing this research question, we consider the following sub-questions that enable the unpacking of the main research question:

- How are the activities of ride-hailing firms shaping and being shaped, in turn, by the socio-cultural political economy structures/dynamics of Ghana’s transport sector?
- What are the outcomes of the intersecting interplays of these global–local interactive processes, and what do they mean for the capacity of ride-hailing to support the generation of decent paid jobs and sustainable use of private cars in the country?
- What could be learnt to affect the push of such technological interventions as the panacea for Africa’s development challenges?

In addition to peer-reviewed sources, the paper relies on evidence from grey literature including media and institutional sources, and empirical sources. Grey literature is an important source of information because they tend to capture the fast-changing dynamics of social phenomena that are not timely documented in scholarly sources. Indeed, media reports, for instance, are increasingly being endorsed in Ghana’s (Mintah et al., 2021; Obeng-Odoom, 2011; Oteng-Ababio and Agyemang, 2015; Boateng, 2016, 2022a, 2022b, Boateng et al., 2021, 2022a, 2022b) and Africa’s scholarly community (see e.g., Yahya, 2006; Boateng and Wright, 2019) as an important source of information for investigating development questions about the country and the continent. And in the specific case of internet-based ride-hailing, since ride-hailing firms rarely share data (for privacy and other reasons) and the veracity of the little they occasionally share too is often difficult to verify independently, researchers have found the media as an invaluable source of information for studying the industry (see e.g. Bialik et al., 2015; Silver and Fischer-Baum, 2015 in Agyemang, 2020).

However, the likelihood of bias reporting implies that care must be taken to consult a variety of media sources to enable a balanced picture of the phenomenon under study. Accordingly, information was sourced from local private (e.g., Myjoyonline) and public (e.g., Graphic Online) as well as international media organizations (e.g., Reuters). The search terms used to assemble the media materials include “internet-based ride-hailing in Ghana”; “Uber in Ghana”; “app-based taxi transport in Ghana” “ride-sharing/sourcing in Ghana”, “vehicle-for-hire in Ghana”, “on-demand ride services in Ghana” on Google and in the search engines of reputable Ghanaian news agencies such as Graphic Online and Myjoyonline. The search terms employed in assembling the media materials from Google and the databases of the news agencies were similar to the terms used in gathering the scholarly materials save that the majority of the academic materials were gathered from more rigorous sources including Google Scholar; Science Direct, JSTOR, Scopus, and ProQuest.

Many of such databases exclude African scholarship (see e.g., Obeng-Odoom, 2019), so after reviewing the initial materials for relevance, purposive techniques and the snowball sampling technique was applied to search and incorporate relevant excluded references. This helped in broadening the scope of the materials search. The institutional materials were sourced from relevant bodies such as the Parliament of Ghana (e.g. Road Traffic Regulations of 2012 (LI 2180); the National Road Safety Authority (e.g. NRSC, 2014), and Uber (e.g. Camp, 2017; Hall and Krueger, 2016)—the major ride-hailing firm in Ghana and globally. The study gathered the empirical data by combining both purposive and snowball sampling techniques—two of the widely regarded qualitative sampling techniques—to engage 40 informants operating in Ghana’s ride-hailing industry in various capacities as drivers, riders, car owners, and researchers.

In gathering the data, the researchers first advertised the study on various social media platforms including Facebook and WhatsApp, inviting eligible informants to participate. After volunteering information, the initial participants were asked to assist with contacts with other potential informants. Through this, the contacts of a total of 59 potential informants (including riders, drivers, and car owners) were received. Upon follow-up, sixteen (16) declined participation. Of the remaining fifty-two (52), seven (7) did not participate fully—as at the time of writing, they had not responded to some follow-up questions sent to them. Their perspectives were, therefore, not included in the final data used for the analysis. Published studies on Ghana’s app-based ride-hailing industry are very limited. Therefore, to gauge how the preliminary findings sat with the observations of the community of scholars on the topic, we developed a second interview guide based on the themes that emerged from our interactions with the riders, drivers, and car owners to engage 4 urban transport scholars who research ride-hailing in the country.

As shown in Table 1, the majority of the riders are females, but all the drivers are males. We found some additional evidence that confirms the dominance of male drivers in the industry. For instance, of the 15 riders who participated in the study, only 1 reported that they had ever met a female driver. The 11 car owners who participated in the study apply a combined total of 41 cars to the delivery of ride-hailing services; their drivers are all males. Some media reports suggest that there were only seven registered *female* Uber drivers in the whole of Ghana as of 2017 (B&FT Online, 2017). Similar gender dynamics apply in relation to the car owners. The overrepresentation of males in our driver and car owner respondents is consistent with the general gender dynamics in Ghana’s ride-hailing industry (see Agyemang, 2020) and the commercial passenger transport sector generally (Boateng, 2020; Boateng et al., 2022; Okraku, 2016).

**Table 1:** Details of participants, fieldwork, and other activities.

Participants		Gender		Mode of engagement
	Number	Male	Female	
Riders	15	4	11	Face-face interviews
Drivers	11	11	0	Face-face interviews; WhatsApp messaging
Car owners	11	10	1	Face-face interviews; WhatsApp messaging
Researchers	4	4	0	Email; Zoom interview; WhatsApp messaging
Total	40			

Source: Authors’ fieldwork.

We limited our consideration of the respondents’ demographic characteristics to gender because other demographic issues have been widely covered in the literature. For instance, multiple systematic studies have confirmed that it’s the younger, highly educated, and relatively high-income-earning class that patronize Ghana’s app-based ride-hailing industry the most (see e.g., Acheampong et al., 2020; Agyemang, 2020; Dzisi et al., 2020). In terms of the medium of data collection, as shown in Table 1, we employed a mixture of semi-structured face-to-face and Zoom interviews; email exchanges; and WhatsApp messaging. The interactions with the first thirty (30) respondents (15 riders, 10 drivers, and 5 car owners) were through face-to-face interviews. The remaining ten (10) were engaged through a combination of the other mediums. The data collection sessions varied from approximately 45 min, and close to 1 h of face-face and Zoom interviews to 3 days or more of email and WhatsApp exchanges. The empirical data, together with the evidence from the grey literature and the few published studies on the topic about Ghana and other African countries and internationally, were weaved together to shed light on how ride-hailing technology is shaping and is, in turn, being shaped by the socio-cultural political economy structures of Ghana’s commercial passenger transport sector, and the implications of the outcomes for the technology’s potential to support the generation of decent paid jobs and sustainable use of private cars in the country.

After an overview of the problem framing, motivation, and sources of data for the study, the rest of the paper is divided into four sections. Section “How ride-hailing is transforming and being transformed, in turn, by Ghana’s transport sector” considers how ride-hailing technology is shaping and being shaped, in turn, by the socio-cultural political economy structures/dynamics of Ghana’s transport sector. Section “Ride-hailing and efficient use of private cars and access to decent paid jobs in Ghana” considers the nature of the transport options and employment opportunities ride-hailing technology avail in Ghana, and what they mean for the positioning of the technology as capable of facilitating efficient use of private cars and access to decent paid jobs. Section “Conclusion” discusses the key highlights of the paper followed by some brief concluding remarks.

## How ride-hailing is transforming and being transformed, in turn, by Ghana’s transport sector

### Transformations in Ghana’s commercial passenger transport sector

Ghana’s public transport sector is dominated by *Tro-Tros* (minibuses). Nonetheless, shared taxis and *Okadas* (motorcycles) also exist. They offer flexible and generally affordable transport services. Concerns, however, exist about the quality of service they provide. Consider the kind of vehicles applied to the delivery of Tro-Tro and shared taxi services or commercial passenger transport services generally in Ghana, for instance. The vehicles are often old and are kept on the road even as they get older and more dangerous (see e.g., Boateng, 2021a; Obeng-Odoom, 2010, 2013). This has earned them various derogatory nicknames including ‘death traps’; ‘safety hazards’; ‘Eurocarcas’; ‘flying coffins’, and ‘moving morgues’ in Ghana and other African countries (see Boateng, 2021b, p. 8).

Generally, ride-hailing firms refuse to register such vehicles to be used for ride-hailing services (even though some riders reported that some ride-hailing vehicles are just as rickety as traditional taxis). Aside from the low vehicle quality, the existing taxi industry and the commercial passenger transport sector generally have other critical challenges. For instance, their fare-charging system is erratic and deeply subjective. Pre-fixed fares exist. Nonetheless, passengers often negotiate fares with drivers when the vehicle is being hired. During such negotiations, drivers just eyeball or guesstimate travel distance or time and, accordingly, determine transport fares. They also frequently determine fares based on their perception of the financial status of potential passengers. As Obeng (The names used are all pseudonyms.), a car owner remarked: ‘If you dress as a pauper, you might get a good discount. If you dress like somebody who is high, an elite, somebody walking around with gadgets and fancy jewelry, the price could be tripled’.

Thus, the pricing system is highly unstandardized, and too discretionary. Ride-hailing metered-pricing system is, however, quite predictable; it eliminates discretion and, therefore, potential abuse or cheating. The participants reported that they even use ride-hailing trip price estimates as benchmarks for negotiating transport fares with traditional cab drivers. Thus, overall, as one car owner, Tetteh, remarked, the emergence of ride-hailing has ‘raised the standards [of taxi service delivery in the country]. They have upped the game in terms of pricing transparency; quality of vehicles and standards of service that riders receive’.

But these are not the only ways ride-hailing technology is transforming Ghana’s commercial passenger transport sector. There are also other transformations related to convenience and security—for both drivers and riders alike. First, to access traditional taxi or *‘Tro-Tro’* (minibus) services in Ghana, commuters must trek or, in emergency situations, run to the nearest terminal or roadside. This is hardly convenient, especially in times of emergency or when one has a lot of heavy luggage and cannot easily move to the roadside or the nearest bus terminal. The drivers too face similar inconveniences as they have to park cars at designated loading stations and wait for passengers or roam the city to search for them, costing time, car depreciation, and fuel.

With the emergence of ride-hailing technology, potential riders can use their smartphones to book rides from any location. The result of this for drivers is that they do not have to waste time and fuel waiting or roaming the cities for passengers. The study participants frequently cited these conveniences as some of the key transformations occasioned by the rise of ride-hailing technology in the country. The submission of Adwoa, a female rider in her mid-twenties, captures the general orientation of the riders as follows:

I felt it was convenient. I mean we are in a technology age, and me having to go down to the roadside or the junction and now stop a taxi or struggling getting transportation whereas I can sit in the comfort of my home or my office and then request a ride and the driver comes, I go wherever I am going. I mean I felt it was convenient. It was not stressful.

The remarks of Razak, a driver in his late twenties, capture the orientation of the drivers on the topic

When you are a taxi driver, you drive empty looking for passengers. But now as Uber driver or Bolt driver, you sit down when the request comes, you go. That’s an advantage; that’s a good thing. Because, for taxi you will roam; you can’t even park. If you park, you are not going to get anyone. So, you will need to roam, and waste the fuel.

Further to convenience, the study participants reported that app-based ride-hailing services are also *affordable* (compared to traditional taxicabs). For instance, Acheampong et al. (2020) found that in Accra, the capital, a typical 25–30 min lone travel in a conventional taxi hailed on the street (known locally as ‘dropping’ or ‘hire’) could cost about GH¢80 ($13.16). But when the same trip is done via a private taxi, it could cost around GH¢21 ($3.82). Shared Tro-Tro is more affordable than both of them; the same trip when done via Tro-Tro will cost an individual just GH¢4 ($0.72). However, unlike Tro-Tro and also traditional taxis, ride-hailing trips are GPS-enabled and, therefore, *traceable*/*trackable* with the result of reducing the risk of harm (for both drivers and riders) during travel and increasing the chances of recovering lost items. As a result, more and more Ghanaians are embracing the technology, with the unintended consequence of fueling a sense of bitterness among the traditional taxi drivers who feel their source of livelihood is being undermined.

All the drivers interviewed for the study reported that they have been involved in some form of confrontation with a traditional taxi driver before, confirming Agyemang (2020)’s finding that Ghana’s online and traditional taxi drivers are engaged in a ‘turf war’ for control over the public transport space. Similar developments elsewhere including in some parts of Africa have led to violent clashes (see e.g., Agyemang, 2020; Kaye-Essien, 2020). For now, Ghana has not witnessed any major problems as the turf war manifests as insults, threats, competition for passengers and road spaces, and other forms antagonism. Overall, the evidence shows that the positive transformations of ride-hailing in Ghana’s road transport sector including increased convenience, affordability, and security in the pursuit and the provision of commercial passenger transport services co-exist and intermingle with other concerning transformations, with potentially deleterious consequences for social order and harmony. Although the transformation in Ghana’s commercial passenger transport sector and society generally by ride-hailing technology is very significant, the dynamics of the transformations are not unidirectional. The Ghanaian commercial passenger transport sector and society generally are also affecting ride-hailing firms to operate in ways that are different from their modus of operandi in their Global North countries of origin. The next sub-section considers one of such important transformations: the adoption of a cash mode of payment

### Transformation in ride-hailing firms’ operation

Ride-hailing in advanced capitalist countries—including the technology’s country of origin—the United States—has become synonymous with never having to pull out your wallet again (Bhattacharya, 2015). Thus, the technology was originally designed to be an entirely cashless experience. This reduces default risks for ride-hailing firms as they take their commissions from drivers immediately after trips are completed. The firms, however, began, to experience penetration problems when they began to launch operations in the cash economies of Global South countries, forcing them to go low-tech by introducing cash payments and adapting their business to suit the exchange structures of the new brand of consumers (for a review, see Bhattacharya, 2015).

Thus, even though the ride-hailing technology was originally designed to be an entirely cashless experience, the use of cash as the main means of exchange in the Global South has meant that the ride-hailing firms spreading into those societies have been forced to tweak the cashless foundation of their business. For instance, in 2015, Uber explained why the company decided to go cash as follows:

We know that cash is still the dominant payment option for millions around the world, especially in emerging markets and smaller cities. These ‘cash’ experiments are really exciting for us, given the success we’ve seen in these test markets and the potential that exists to take this even further into more cities around the world (Bhattacharya, 2015).

Thus, the ride-hailing firms are being forced to adapt their operations to the contextual peculiarities of recipient societies in ways that are different from how they operate in their countries of origin. As indicated elsewhere, as of 2018, Uber alone was said to have about 180,000 active riders in Ghana and some 3000 driver-partners as of 2016 (Dahir, 2017). It is doubtful that ride-hailing firms will have enjoyed such high public acceptance in Ghana if they did not tweak the cashless foundation of their business as some drivers cancel trips when they realize that the potential rider intends to pay with a credit card. The submission of Adwoa, a female rider in her mid-twenties, illustrates the development:

I requested an Uber, the driver realized it was a card trip, he canceled. I re-requested, he accepted, realized it was a card trip, he canceled, and I didn’t request again. That was for one particular trip. The second trip, the Uber driver actually showed up, he realized it was a card trip, he said he won’t go, he canceled and moved on.

The drivers explained that they decline credit card trips because such trips allow the ride-hailing companies to withhold their sales for a long time. This, they complained, leave them with limited working capital to finance their operations including purchasing fuel and paying for maintenance. The complaint of Ackom, a driver in his late 20s, aptly illustrates the drivers’ issues with credit card trips.

The point is you are buying fuel, you are servicing your car and you need cash [to do that]. With the experiences that I have had with the ride-hailing firms, you don’t receive your sales until a period of time. So, what happens if I don’t have money to fuel the car or if I don’t have money to service the car until then?

Carmody and Fortuin (2019) have reported similar complaints by online drivers in South Africa. Overall, the evidence considered shows that the ride-hailing firms operating in Ghana have tweaked their business model to suit Ghana’s cash-dominant economy in ways that allow urban dwellers to apply the technology to access an alternative to existing modes of commercial passenger transport as riders, and jobs as drivers. Thus, the technology is helping them to solve some of the continuing urban transport and unemployment problems they face in the country. But what is the nature of the transport options and employment opportunities that ride-hailing avail in the country, and what do they mean for the positioning of the technology as capable of facilitating efficient use of private vehicles and access to decent paid jobs? The remainder of the paper addresses these issues.

## Ride-hailing and efficient use of private vehicles and access to decent paid jobs in Ghana

### Efficient private vehicle use

Ride-hailing is often presented as a promising platform for boosting efficient and flexible use of private vehicles by providing *‘a need-basis’* access to—instead of ownership of—private vehicles (Alonso-Moraa et al., 2017; Cohen and Kietzmann, 2014; Fulton et al., 2017; Jin et al., 2018; Santi et al., 2014; Vazifeh et al., 2018). Ultimately, consumer behavior will be altered towards possessing fewer private vehicles, and an overall reduction in the number of vehicles on roads and lead to increased public health benefits in reduced congestion, energy consumption, greenhouse gas (GHG) emissions, and car crashes. Our findings from Ghana just as much corroborate as they challenge the discourses on ride-hailing and sustainable use of private vehicles.

We found some evidence that corroborates the claim that ride-hailing facilitates the sharing of private vehicles. Afia, a rider in her mid-20s reported, for instance, that ‘even though I do not have a car, Uber makes me mobile’. Tetteh, a car owner in his late twenties, made similar remarks:

They [referring to ride-hailing firms) have given people the chance to ride in a vehicle that they do not own. You have almost full access to services of any typical owner. You do not have to own a car to have access to a private ride.

However, most of the vehicles used to provide ride-hailing services in Ghana, mainly Daewoo Matiz; Hyundai i10/Eon/Getz; Kia Picanto/Morning; Toyota Yaris/Vitz/Echo/Platz and Suzuki Alto, are specifically imported for that purpose. Thus, very few people *‘convert’* or commercialize existing private vehicles. For instance, the 11 drivers and 11 car owners who participated in the study supply a combined total of 41 private vehicles for the delivery of ride-hailing services. Only 4 were, hitherto, private vehicles commercialized for ride-hailing services—the remaining 37 were imported for the express purpose of providing ride-hailing services. This implies that the *kind of private vehicle-sharing* ride-hailing facilitates in Ghana does not lead to overall reduction in the number of vehicles on the roads; it actually increases them—manifesting as increased importation of more and more private vehicles for ride-hailing business in the country.

But that is not the only way ride-hailing is contributing to the inundation of Ghana’s roads with more private vehicles. There are others. Our findings corroborate that of Acheampong (2021) and Acheampong et al. (2020) that one of the critical factors that makes ride-hailing appealing to Ghanaians is *privacy*—the opportunity to have exclusive use of vehicles instead of sharing them with other riders. For instance, explaining why she prefers ride-hailing services to *Tro-Tro*, the ubiquitous minibuses that serve the majority of Ghana’s urban population, Akosua, a female rider in her late 20s who has been patronizing ride-hailing service since its emergence in Ghana, submitted that

I really hate it [Tro-Tro], I am being truthful. I really hate Tro-Tro. Especially when you are tired from work and you want to come home and then you are in the big one –the 4-seater, and then you have somebody who is drunk seated beside you and then sleeping on your lap and those things. You’ve had enough. You’ve had a long day. So, Uber gives you your privacy sort of. I can sit in the car and take off my shoes, my wig is off.

The downside of this, however, is that it incentivizes lone travel and, thus, undermines ride or trip-sharing. Overall, the rise of ride-hailing in Ghana is not just driving a shift from shared public transport options; it is also propelling increased use of private vehicles which stay at a lower passenger occupancy rate while on the road. The result of this has been a worsening state of traffic congestion in the cities, the cost of which riders are already paying in increased travel time, and increased share of income spent on transport—as illustrated by the extracts below.

I remember taking an Uber from Dansoman to Oyarifa [both are suburbs of Accra]. I think the price was about GH¢40. On my way home, there was so much traffic. I told the driver I will want to end the trip because I am sure by the time I get home, the price will be just too much for me to pay. I just ended the trip. I was less than halfway through it, but I had to pay more than half of the price Uber showed at the beginning of the trip. And I think that was really worrying because if I had stayed through the trip, I would have had to pay way more than I had actually budgeted (Adwoa, a female rider in her mid-twenties).

You will pick an Uber and then you will be stuck in traffic and then you know you are going to pay GH¢20, you get to the place, and you are paying GH¢200, GH¢500 (Akosua, a female rider in her late 20s).

I think it has exacerbated the traffic situation. You already have a traffic congestion plague in this country and Uber has just quadrupled it. So negatively, it has affected people’s pocket because you end up sitting in the car the whole time and the amount of money you are going to pay increases because of your trip time (Tetteh, a car owner in his late twenties).

The problems with ride-hailing-inspired importation and lower passenger occupancy rate of private vehicles in Ghana are not limited to increased travel time and share of income spent on transport due to congestion. There is also the issue of pollution. The International Trade Administration of the US Department of Commerce data suggests that over 90% of Ghana’s vehicle imports, including those used for ride-hailing services, are used vehicles (ITA, 2020). Used vehicles are often not just prone to malfunctioning and, therefore, crashes, they also are usually highly polluting (see e.g., UNEP, 2020). By stimulating the *importation* and *usage* of more used private vehicles, the activities of ride-hailing firms could end up worsening the already troubling issue of vehicular pollution in the country (see Amegah et al., 2021; NRSC, 2014; Obeng-Odoom, 2010; 2013; USAID, 2016).

These environmental and other concerns from Ghana align with the growing wave of challenges against the environmental and public health benefits often claimed on behalf of the ride-hailing technology (see e.g., Aytes, 2012; Blosfield, 2021; Ettlinger, 2016; Jin et al., 2018; Rosenblat, 2018; Diao et al., 2021; Erhardt et al., 2019; Houeland, 2018). It is, however, not just its traffic congestion and vehicular pollution reduction credentials, the technology’s capacity to support the provision and access to decent paid jobs too is increasingly being questioned in several parts of the world (see Woodcock and Graham, 2020, for instance). The next section considers Ghana’s situation.

### Ride-hailing and access to decent paid jobs in Ghana

Uber sold its business to the Ghanaian government as a platform for creating *‘thousands of economic opportunities for Ghanaians’* (Kaye-Essien, 2020, p. 724). We sought to assess the nature and conditions of the economic opportunities the company and for that matter, ride-hailing firms’ operations generally have created or are creating for Ghanaians—with particular focus on car owners and drivers. Ride-hailing firms have long argued that they create opportunities for *‘independent mini entrepreneurs* to profitably commercialize their private cars (Knox, 2019; Blosfield, 2021; Bond, 2020). This claim is empirically verifiable in Ghana. All the car owners who participated in the study confirmed that the ride-hailing industry is a profitable entrepreneurial venture. For instance, Tetteh, a car owner in his late twenties, submitted that

I like it [ride-hailing business] because of the frequency of income. Other investments that we have in the country, for example treasury bills: Your money is stuck for the term of the investments, minimum of 91 days. You cannot use it for any other reason. You have to make sure it’s there till it realizes the interest on it. But with Uber because you keep getting the payment in weekly installments, you are able to put it into other investments. So, you have more liquidity which you can use to unlock further investments. So, you can make much more than the percentage you typically make on treasury bills because you have more access to your money, and you can move it around to make more money for the same amount of time. So, it’s definitely a better investment than your other typical investment.

Asamoa, another car owner in his late thirties, made similar remarks.

Uber has made a great impact. I have been able to make extra income. So, monies I used to spend from my main job on certain things, I don’t spend those monies on those things anymore. I could use monies I get from Uber for other expenses. It’s helped me a lot. Other projects I am planning on working on, I am getting extra cash for it. It’s changed a lot. It’s helping me to do things I want to do easily.

The problem, however, is that very few Ghanaians operate in the online ride-hailing industry as self-employed *vehicle-owning entrepreneurs*. The majority are *casual labors* who sell their driving skills for a living under either ‘sales or work and pay’ contract arrangements with car owners. Drivers employed under *sales contracts* operate their car as a sort of daily franchise, for which the owner, depending on the contract terms, demands a daily or weekly fee (popularly called ‘sales’). The daily or weekly return for the driver is what remains once the ‘sales’ has been made and the cost of operation (e.g., ride-hailing firms’ fees; fuel and internet costs)—towards which car owners do not contribute—have been deducted. Under the *work and pay contract system*, the driver operates the car and pays the owner a weekly or monthly sum up to a pre-agreed vehicle value, after which ownership of the vehicle transfers to the driver. Essentially, the driver amortizes a certain weekly or monthly payment and, takes possession of the car when the payments reach the full value.

Our data show that the standard practice on the Ghanaian market, in relation to the *work and pay contract system*, is that car owners often double the price of the car for drivers. Thus, the driver operates the car for the owner to earn 100% profit on the cost of the car before s/he earns ownership rights. Car owners, under both *work and pay* and *sales contract* systems, normally demand weekly returns of GH¢ 400–500 ($67–$84) (The Dollar–Cedi exchange rate on August 3, 2021) from drivers. This excludes ride-hailing firms’ commissions/charges (which also range from 15% to 25% of the value of each trip); operational cost (e.g., fuel, internet), and depending on the contract terms, maintenance. How these conditions, together with the ride-hailing companies’ business model generally, shape drivers’ behavior requires careful analysis.

When all is said and done, the only time any financial value is generated in the ride-hailing industry is when a driver picks a rider and receives a payment for the service. The income generated from this process is what covers all the costs—vehicle, sales, fuel, internet, maintenance, taxes, and the ride-hailing firms’ commissions—that make the existence of the ride-hailing industry possible. Ride-hailing companies’ business model, however, fundamentally involves avoiding sales taxes, the cost of vehicles, repairs, insurance, and meeting obligations for social security and health benefits for their drivers. They have long objected to classifying their drivers as their *‘employees’* (Knox, 2019; Blosfield, 2021; Bond, 2020; Woodcock and Graham, 2020). Further, there are no labor protections in Ghana for drivers to assert the benefits ride-hailing companies strenuously strive to evade from their ‘*other employers’*: their car owners (Boateng, 2020, 2021b). The result of these is immense pressure on drivers to generate as much revenue as possible to cover not just all the costs that make the existence of the ride-hailing industry possible, but also their own personal wellbeing and that of their families. These pressures invariably incentivize unhealthy driving practices. The participants’ accounts suggest that

Some drivers don’t even go home; they have their toothbrush, sponge, and towel in their car. They drive from morning to evening, park at filling stations, take a nap, take a bath and continue. Their wives see them, once a month. Yeah, it’s happening, once a month. If I take you round, there are some areas you will see a lot of Uber cars parked. They are asleep. The drivers are asleep. They are tired. They can’t go home, not that they don’t have homes, they have, but they can’t go home. Most of them do work and pay. Their car owners are demanding [sales]. (Ackom, a driver in his late twenties).

In addition to their precarious working conditions, the manner the ride-hailing firms structure the drivers’ work processes also incentivizes unhealthy driving behavior. The laws of Ghana, specifically section 118 of the Road Traffic Regulations of 2012 (LI 2180), proscribe commercial drivers from driving ‘for a continuous period exceeding four hours; or for a period amounting in the aggregate to more than eight hours in a period of twenty-four hours’. The ride-hailing companies, however, require drivers to take a break only when they have worked for 12 h, which clearly violates the law. Nonetheless, this violation is left unchecked and unenforced by regulators. Also, instead of honoring drivers’ consistent complaints for a reduction in what they unanimously described as *‘*very high commissions/fees’, the ride-hailing firms rather give them promotional offers requiring them to complete a set number of trips within a certain amount of time to earn extra money. This incentivizes unhealthy driving behaviors such as overspeeding as the drivers rush to complete as many trips as possible to be eligible for the offers.

Multiple systematic studies have shown that similar precarious work processes and incentives are what drive reckless and aggressive driving behaviors among minibus (*Tro-Tro*) drivers which often lead to crashes and other unsafe driving outcomes in the country (see e.g., Boateng, 2021c, 2021d; Dotse et al., 2019; Obeng-Odoom, 2013). It is difficult to assess how these unsafe driving outcomes also play out in the ride-hailing industry because road crash data in Ghana are currently not disaggregated in terms of how much are caused by ride-hailing vehicles. However, media reports suggest that the safety records of such cars are increasingly becoming concerning (see e.g., Ghanaweb, 2019; Modern Ghana, 2021). These concerns have even moved some ride-hailing companies to introduce accident insurance in the country (see e.g., Ghanaweb, 2018).

Finally, the drivers and the car owners’ accounts suggest that profit margins in the ride-hailing industry are gradually tailing off. The reasons for this include growing security concerns including reports of kidnaping of riders and murder of drivers (see e.g., Graphic Online, 2019b; Ghanaweb, 2021). Other reasons include rising ride-hailing companies high charges/commissions; apparent saturation resulting from the increased number of drivers and car owners trooping into the industry, and traffic congestion which increases travel time, and, therefore, cost. The participants’ accounts suggest that the intersecting interplay of these web of factors are undermining ridership and, therefore, profit margins. These developments have concerning implications for the livelihoods of the many casual drivers who are now braving the wrath of traditional taxi drivers and literally *slaving* themselves under ‘work and pay contracts’ with the hope of becoming ‘self-employed car-owning entrepreneurs’ in the future. The industry might not be as profitable by the time they may achieve their dreams.

## Discussion

The rise of ride-hailing in Ghana is transforming the commercial passenger sector in diverse ways. For instance, the metered pricing system, despite transparency concerns (see Acheampong, 2021; Agyemang, 2020), enforces some level of predictability in contrast to the existing one founded on the bargaining skills of drivers and passengers. Price surges and gridlocks could increase the trip costs. Nevertheless, ride-hailing trips are generally seen as affordable compared to hiring traditional taxicabs. The traceability of ride-hailing trips is perceived to reduce the risk of harm during travel and increase the chances of recovering lost items or tracing harms/crimes and criminals. Finally, the vehicles which bring the services to riders’ doorsteps tend to be fairly new and are kept in good condition often with air-conditioning even though concerns exist that some of them are just as rickety as the traditional taxis or *Tro-Tros*.

Further to driving up the standards of road transport experience, ride-hailing has opened up economic opportunities in Ghanaian society. It has created opportunities for people to monetize their driving skills, even if they have to brave the wrath of traditional taxi drivers. As of 2018, Uber alone reportedly had over 3000 active partner drivers in the country (see Agyemang, 2020, p. 65). Also, those who have access to surplus income to acquire private vehicles for ride-hailing services are making great returns on their investments. For instance, it has been shown that under the work and pay contract system, car owners could realize a 100% return on their investment within a short period of 2 years.

However, not only do these positive outcomes impact differently on various classes of people, but they also co-exist and intermingle with other concerning outcomes that question ride-hailing potential to support fair labor remuneration, equitable access to transport, public health, climate, and general development goals in the country. The drivers of these outcomes vary, as detailed below: some are built into ride-hailing firms’ *business model* (e.g., its founding on smartphone technology); others are *cultural* (e.g., Ghanaians’ preferred way of experiencing ride-hailing services); some of them too are *structural* (related to the power relations underlying Ghana’s passenger transport sector).

Multiple studies have confirmed that the benefits of ride-hailing in Ghana are enjoyed almost exclusively by the younger, highly educated, and relatively high-income-earning class (Acheampong et al., 2020; Agyemang, 2020; Dzisi et al., 2020). This outcome is just as much shaped by the nature of the ride-hailing business model as local cultural factors. First, one must be conversant with technology to be able to apply smartphones to access ride-hailing services—a skill that comes in handy for young and educated people. Second, it is only recently that some ride-hailing firms have begun experimenting with trip-sharing options in Ghana (e.g., *UberXShare*) where unacquainted passengers with similar origins and destinations could share a ride and split the fare. The experiment is, however, poorly patronized, which is not surprising given the widespread unwillingness among ride-hailing consumers to share rides with strangers in the country (see e.g., Acheampong et al., 2020; Acheampong 2021). Indeed, as shown in this study, the opportunity to have exclusive use of vehicles instead of sharing them with other riders is one of the key influences of ride-hailing adoption in Ghana.

The result of the founding of ride-hailing services on smartphone technology and Ghanaian riders’ taste for lone travels is that older and uneducated people who are not tech-savvy and low-income people without the financial wherewithal to afford smartphones and lone-travels are excluded from the benefits of ride-hailing technology in the country. But age, education, income, and lone-travel culture are not the only pathways shaping unequal access to the benefits of ride-hailing technology in Ghana. There is also a gender dimension. The study’s findings corroborate that of prior research (e.g., Acheampong et al., 2020; Acheampong 2021; Agyemang, 2020; Dzisi et al., 2020, 2021) that women highly patronize ride-hailing services in Ghana–as riders. Yet, males dominate the industry as drivers and car owners. Essentially, while women spend meaningfully in the industry as consumers, opportunities for them to directly benefit financially from it as service providers are severely limited. But this dynamic is not a peculiar feature of the ride-hailing industry; the industry has only patterned after the existing strictures of male-dominated gender power structures embedded in Ghana’s commercial passenger transport sector (see Boateng, 2020; Okraku, 2016).

Another key outcome patterned after the existing power structures underlying Ghana’s commercial passenger transport sector is the lopsided/exploitative power relations between drivers on the one hand and ride-hailing firms and car owners on the other hand. In Ghana, there exists an unmet demand for adequate public transport (see e.g., Oteng-Ababio and Agyemang, 2012, 2015) and continuing decline in opportunities for wage employment (Obeng-Odoom, 2013; Boateng, 2021a; Gillespie, 2017). As pertaining to other African countries (see e.g. Behrens et al., 2016; Klopp, 2021; Klopp and Mitullah, 2016; Klopp et al., 2019; Rizzo, 2011, 2017), whereas local private transport owners/operators have helpfully stepped in to fill these public service gaps, the continuing decline in opportunities for wage employment and the lack of labor rights protections especially in the informal sector create room for them to exploit the many young people who enter the commercial passenger transport sector looking for jobs as drivers.

The result of this is that the drivers sign exploitative contracts with car owners, which they fulfill by being hyper-competitive, aggressive, and reckless on the roads (see Boateng, 2021e, 2021f; Dotse et al., 2019; Obeng-Odoom, 2010, 2013). The evidence considered shows that similar exploitative power relations exist in the ride-hailing industry as well. There are growing concerns in advanced capitalist societies that the advent of ride-hailing firms is generating a race to the bottom, creating a new, digital generation of sweatshops marked by poor working conditions—low wages, minimal job security, and benefits (Aytes, 2012; Blosfield, 2021; Ettlinger, 2016; Jin et al., 2018; Rosenblat, 2018). Some analysts and media reports suggest that similar developments have emerged in Africa (see e.g., Carmody and Fortuin, 2019; Houeland, 2018; 2018; Graphic Online, 2017). However, the evidence from Ghana suggests that the challenge in Africa is not really about ride-hailing firms driving a race to the bottom.

It seems more precise to say that the continent presents a political economy environment perfectly suited for the firms’ business model of profiting from unmet demand for adequate public transport while avoiding the cost of vehicles, their maintenance and insurance, minimum wage, social security, health, and unemployment benefits and other similar obligations. Thus, the emergence of ride-hailing in Africa and the underlying costs and potential for exploitation need to be understood in the context of the continuing deprioritizing of investments in organized public transport, the generation of adequate secure jobs and labor rights protections in the continent, thereby creating room for international (and a few indigenous) ride-hailing firms to profit from vehicles they do not own, and the labor of drivers they do not ‘employ’.

This insight is timely and important because, as shown in this and other studies, in Ghana and a range of African cities today, instead of demanding a sharper policy focus on investing in public transport, the generation of more secure jobs and labor protections—i.e. solutions to the structural conditions that make them vulnerable to the exploitative and oppressive accumulation of powerful actors such as ride-hailing firms—the many young people being heavily exploited as casual labors in the commercial passenger transport sector are locked up in recriminations, fighting each other, as ‘online’ and ‘traditional’ drivers, as the cause of their miseries. Taken together, while specifically focused on the environmental and other costs of the transportation and employment benefits of ride-hailing technology in Ghana, the paper’s overall findings raise broader concerns about the hailing of technological interventions as though they are the magic bullets for socio-economic transformation in Africa (see e.g., Chakravorti and Chaturvedi, 2019; Echendu and Okafor, 2021). The lessons from the rise of ride-hailing in Ghana suggest that while such exclusively technical solutions tend to appear ‘smart’, they easily could take root and pattern after existing strictures of unjust or lopsided power structures in ways that could exacerbate the social and environmental problems they are supposed to address.

## Conclusion

This study has presented the double-faced outcomes of the rise of ride-hailing technology in an African context, showing how it is shaping and being shaped by the local socio-cultural political economy structures/dynamics of the transport sector. While the local contexts have compelled ride-hailing firms to make some notable adaptions to their business model–particularly in the adoption of cash as the main mode of payment—the way ride-hailing firms are shaping the transport sector in Ghana is comparatively more significant. This paper has highlighted the costs of the benefits ride-hailing technology is touted to be contributing to sustainable development goals in the country. If anything, the gains are uneven and are exacerbating existing inequalities between those who control sources of capital/technology and those who do not. At the level of evidence, ride-hailing firms and entrepreneurial (male) car owners are the big winners, while the broader society pays for the environmental and other costs of their private gains related to unhealthy driving practices; increased congestion; shifts from shared public transport, and inundation of the roads with more private vehicles.

While it will be imprecise to say that the private gains of ride-hailing outstrip the public costs and, therefore, the technology is detrimental to Ghana’s development, the considered evidence raises concerns about how the technology’s potential to support climate and other development goals is often framed, raising the need for researchers, policymakers, and regulators to continue scrutinizing the activities of ride-hailing firms in Africa. The small sample size of 40 limits the extent to which the findings could be generalized. Nonetheless, overall, the study brings to the fore strongly the important lesson that dismantling the strictures of unjust power structures underlying Africa’s urban challenges will require more than splashing *‘*smart’ apps and other tech wizardries around. One key issue that bears watching is the future impact of ride-hailing technology on the traditional taxi industry. Will it eventually swallow the industry? Or will the industry eventually adapt some of the technology’s endearing features to defend its position in Africa’s commercial passenger transport sector and what will that mean for fair labor practices and equitable access to sustainable transport and other development goals? We recommend for future studies to consider these questions and their corollaries.

## Data Availability

The dataset generated during the study is available upon request.
